# Integrating Healthcare Services for Indigenous Australian Students at Boarding Schools: A Mixed-Methods Sequential Explanatory Study

**DOI:** 10.5334/ijic.4669

**Published:** 2020-03-09

**Authors:** Janya McCalman, Erika Langham, Tessa Benveniste, Mark Wenitong, Katrina Rutherford, Amelia Britton, Richard Stewart, Roxanne Bainbridge

**Affiliations:** 1 Central Queensland University, AU; 2 Apunipima Cape York Health Council, AU; 3 James Cook University, AU

**Keywords:** Indigenous, adolescents, integrated care, primary healthcare, remote, schools

## Abstract

**Introduction:**

Many Aboriginal and Torres Strait Islander Australian adolescents from remote communities attend boarding schools, requiring integrated healthcare between home and schools. This study explored students’ health status, healthcare service use and satisfaction.

**Methodology:**

A two-phased mixed-methods explanatory design was implemented. 32 Indigenous primary and 188 secondary boarding school students were asked their health status, psychological distress, use of healthcare services in community and boarding school, and service satisfaction. Results were fed back to students, parents and community members, and education and healthcare staff to elicit further explanation and interpretation.

**Results:**

In the previous year, 75% of primary and 81% of secondary boarding school students had visited a doctor. More than 90% were satisfied with healthcare services used. Despite 27.1% reporting high psychological distress, students did not perceive distress as reducing their overall health, nor was distress associated with mental healthcare service use.

**Discussion:**

Despite high levels of service use and satisfaction, this study highlighted the need for improved healthcare integration for Indigenous adolescents between school-based and remote community services. Further research is needed to identify students’ expectations and models for healthcare integration.

**Conclusion:**

With resourcing, schools could play a greater role in facilitating access to healthcare.

## Introduction

Primary healthcare service providers are able to influence and support the development of Aboriginal and Torres Strait Islander (hereafter respectfully termed Indigenous) Australian adolescents’ lifestyles and behaviours at a life stage when many important health risk and protective factors for later life either emerge or strengthen. This opportunity is particularly important given the young age structure of Australia’s Indigenous population, with 37.3% of Indigenous Australians aged 10–24 years [1], and their poorer health status compared to other young Australians [2]. Healthy lifestyle behaviours developed in adolescence have the potential to persist through adulthood and inter-generationally. Furthermore, good health and wellbeing provide a foundation for educational participation and achievement, the sequelae of which contribute as a lifetime determinant of health [3].

Indigenous adolescents who live in remote communities, however, have poorer access to healthcare services than those living in non-remote areas [4,5]. For the approximately 5000 Indigenous remote-dwelling adolescents nationally attend boarding schools for up to 40 weeks of the year for their secondary education [6], access to healthcare services becomes more complicated because they require healthcare provision both in home community and school locations. This research study investigates the extent to which north Queensland Indigenous remote primary school and secondary boarding school students use healthcare services in their home community and while at schools, their levels of satisfaction with these healthcare services, and the extent to which integration occurs between community and school-based primary healthcare and mental healthcare services. Integration is considered as coherent service delivery and care designed to create connectivity, alignment and collaboration within and between services. The goal of integration is to: “enhance quality of care and quality of life, consumer satisfaction and system efficiency for patients … cutting across multiple services, providers and settings. [Where] the result of such multi-pronged efforts to promote integration [leads to] the benefit of patient groups [the outcome can be] called ‘integrated care’” [7].

A recommended level of Australian health assessment for Indigenous adolescents has been established in the National Guide to a Preventive Health Assessment for Aboriginal and Torres Strait Islander People [8]. This states that Indigenous adolescents should be assessed annually for smoking status, body mass index, physical activity, vision, hearing loss (<15 years), oral health and asthma [8]. It also recommended that those aged 12 years and over be offered annual screening for gambling, social and emotional wellbeing, at-risk sexual behaviours, and risk factors for illicit drug use, nutrition and waist circumference, and those aged 15 years and over, for alcohol use. Additionally, the guidelines recommend routine immunisations along with lifestyle risk factor counselling and condition-specific management and treatments. However only 22% of young people received a Medicare-subsidised annual Indigenous-specific health check in 2016, despite these being widely available [4].

Two thirds (66.4%) of the 1,594 Indigenous respondents (15–19 years) to Mission Australia’s Youth Survey 2018 placed a high value upon physical health, and 61.1% on mental health [9]. Coping with stress was the top issue of concern, with 37% of Indigenous respondents indicating that they were extremely or very concerned, and 30% were either extremely or very concerned about mental health [9]. But the most recent Australian Aboriginal and Torres Strait Islander Health Survey (2012–13) found that 11% of Indigenous youth (aged 10–24) did not see a doctor when they needed to, 7% did not see another health professional (nurses, health workers) and 16% did not see the dentist [4]. For young Indigenous people who did not go to the doctor when needed, the most common reasons were: deciding not to seek care (32%), too busy (with work, personal life or family) (28%), waiting time was too long or service was not available at time required (21%), and cost (14%) [4]. Similarly, there is evidence that many Indigenous young people do not seek support for mental and behavioural disorders through either primary healthcare services or hospitals [10].

Adolescence is usually a life stage of generally good health, but remote-dwelling Indigenous adolescents experience the highest burden of ill-health of any Australian adolescent population [11,12]. In 2011, 62% of Indigenous adolescents (10–24 years) had a health condition lasting or expected to last 6 months or more, and 21% reported having been hurt or in an accident in the last 4 weeks [5]. In 2011, the leading contributors to the national burden of disease for Indigenous youth (aged 10–24) were suicide and self-inflicted injuries (13%), anxiety disorders (8%), alcohol use disorders (7%) and road traffic accidents (6%); all of which are preventable [4,12]. Additionally, 39% of remote-dwelling Indigenous youth (15–24 years) experienced high to very high levels of psychological distress compared to those in non-remote areas (31%) [4]. Despite these rates of disease and injury, Indigenous adolescents assess their own health status highly. In 2014–15, 63% of Indigenous young people (10–24 years) assessed their health as either excellent or very good [4]. This apparent disconnect means that distressed or unwell Indigenous adolescents may not perceive a need for, nor seek healthcare.

The reluctance of many Indigenous adolescents to attend a healthcare service or professional when needed, and their high burden of preventable ill-health, suggests that the current healthcare system does not respond well to the needs of Indigenous adolescents [13]. The continuing disadvantage faced by Indigenous adolescents not only in health, but also in education prompted the National Indigenous Health Equality Council’s roundtable to recommend that responsiveness could be improved through integration of healthcare with schools [13,14]. International studies have found that school-based (delivered on school grounds) and school-linked (coordinated at the school but delivered off campus) healthcare offers potential benefits [15]. These include improved: 1) access to healthcare for those rural- or remote-dwelling students who do not otherwise have reasonable access; 2) classroom time due to reduced time lost in travel; 3) follow-up compliance; 4) non-traditional options to healthcare; and 5) collaboration between clinicians and school staff [15].

Many generalist international and Australian adult client satisfaction surveys have found satisfaction rates with healthcare services that exceed 90% [16,17]. There is no published data about Indigenous adolescent’s levels of satisfaction, although almost half of the 1,594 Indigenous respondents (15–19 years) to Mission Australia’s Youth Survey 2018 indicated that they would turn to a general practitioner (GP) or health professional (48.4%) as a source of help with important issues. Similar proportions of Indigenous female and male young respondents indicated their GP or health professional to be a source of support [9]. Generically, satisfaction with a healthcare service reflects a person’s perception of care received and their personal and cultural preferences and expectations. The most important criteria documented for all (mainstream) adult clients are (1) professional competence, and (2) communication and empathy in the patient/health professional relationship [16]. Satisfaction with service provision can improve youth resilience and wellbeing, and behaviours such as engagement and participation in school and community [18,19]. However, there is evidence that Indigenous people perceive many services to be culturally unsafe and inappropriately adapted to respond to Indigenous needs [20,21].

This study focuses on the health, healthcare service use and satisfaction of a cohort of Indigenous remote-dwelling north Queensland students who are required to attend boarding schools for secondary education because there is limited or no secondary schooling available in their home communities. It forms part of a broader ecological study to support and enhance the resilience of these students; other papers have provided a study protocol [22], descriptions of the survey development and validation [23,24], description of staff capacity development [25] and student survey baseline results [26]. Anecdotal evidence (2016) attained through this broader resilience study suggested that some students had untreated health conditions upon arrival at or during boarding school education, and/or experienced patchy care between remote home community and school-based healthcare services. The intent of the current study was to establish a baseline understanding of students’ levels of and satisfaction with healthcare service use to inform efforts by primary healthcare services and boarding schools to improve the availability, use, and integration of healthcare services by remote-dwelling Indigenous students, and students’ satisfaction with service use.

The research questions were: To what extent does the cohort of remote dwelling Indigenous students: 1) assess their health status as excellent or very good; 2) use (dis) integrated healthcare services through their schools compared to home community services, and mental healthcare and/or wellbeing services; and 3) express satisfaction with the healthcare services they receive? 4) What are the implications of these findings for integrating healthcare services across remote home community and education settings. The study is guided by three hypotheses:

1. Consistent with the national finding that 63% of Indigenous young people assessed their health as excellent or very good [4], we hypothesise that the majority of this cohort of remote-dwelling Indigenous students will assess their health as either excellent or very good;
2. Given that students are in school 39/52 weeks per year and that access to healthcare service is higher in regional or urban (boarding school) locations than in remote communities nationally, we hypothesise that more secondary boarding students will access healthcare services through schools than in home communities; and
3. Since home community-based healthcare services are likely to be more familiar with Indigenous health issues and family healthcare preferences; we hypothesise that students’ satisfaction with home community-based healthcare services is likely to be higher than that with school-based healthcare services.

## Methodology

This baseline research to identify Indigenous adolescents’ access to, use of and satisfaction with health services was invited and supported by Apunipima Cape York Health Council, the community controlled Indigenous health service that provides comprehensive primary healthcare to Cape York families. All authors participated in the design, implementation and write up of this research; one (MW) is an Indigenous public health practitioner, one (RB) an Indigenous public health researcher, three (KR, AB and RS) are non-Indigenous education practitioners, and three (JM, EL and TB) non-Indigenous public health researchers. The research has ethical clearance from James Cook University Human Research Ethics Committee (H5964), Central Queensland University Research Ethics Committee (H16/01-008), and Department of Education, Queensland Government (File No. 50/27/1646).

Resilience is considered as an ecological concept whereby in the context of exposure to significant adversity, the student navigates

“their way to the psychological, social, cultural, and physical resources that sustain their well-being, and their capacity individually and collectively to negotiate for these resources to be provided in culturally meaningful ways” [27,28].

Healthcare services are one of the resources that students navigate towards (along with peer and family support, education, and other social services) in order to sustain their wellbeing. Consistent with the Indigenous view of health [29], in this study, we considered wellbeing as a holistic concept that includes students’ physical, social, emotional, cultural, spiritual and ecological wellbeing.

### Study design

The study used a mixed-methods explanatory design where quantitative survey data and qualitative focus group data were both used to capture the trends and details of students’ health service use and satisfaction. Together, the quantitative and qualitative methods allowed for a more complete and robust analysis of the complexities of health service use and satisfaction by Indigenous boarding school students, that encompassed the strengths of each method [30]. The design entailed two consecutive phases within one study: collecting and analysing quantitative and then qualitative data [30].

### Context

**Figure 1:**
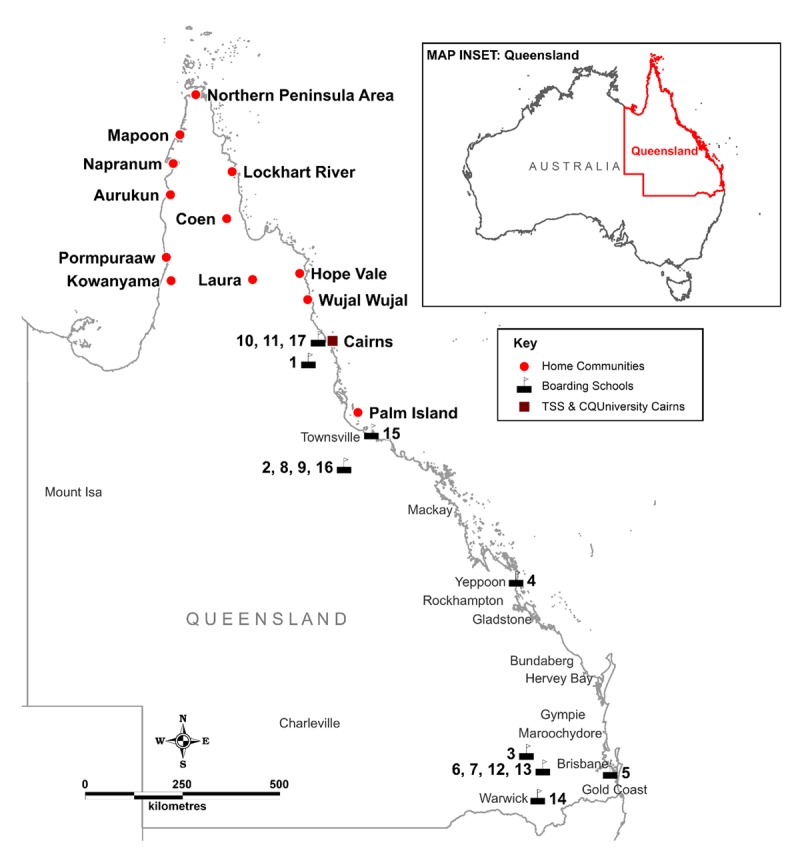
Map of Cape York and Palm Island communities, and destination boarding schools.

Students who participated in this study came from 12 remote and very remote communities on Cape York and Palm Island, Queensland (Figure 1). The Cape York communities were formed from the late 1800s to early 1900s as result of the removal and relocation of Indigenous people to mission settlements. Each community has a distinctive history, tribal population groups and church influence. Currently, there are 5846 Indigenous residents of Cape York; families tend to be large and extended, and the population is young [1]. The burden of ill-health and health risk factors in Cape York Indigenous communities is high, with a low median age of death (58 years) and high rates of smoking, obesity, risky alcohol consumption, cancer, injury, potentially preventable hospitalisation, and avoidable death compared to other Queensland health and hospital service regions [31].

In north Queensland, primary healthcare services are not resourced to provide specific youth-targeted services, and there are few youth-specific specialist fly in, fly out healthcare services. Queensland Health provides 10 primary healthcare services, and two multipurpose healthcare services in Cape York as well as fly-in fly-out teams and visiting specialist services [32]. The community-controlled Apunipima Cape York Health Council provides comprehensive primary healthcare to eleven communities via its healthy lifestyles, health promotion and family health (maternal, child and school health) teams. GP services and social and emotional wellbeing support is provided through Wellbeing Centres in four communities and SEWB teams in the other seven [33]. Non-government providers such as the Royal Flying Doctor Service provide emergency retrieval as well as primary healthcare clinics incorporating general practice, child and family health, Indigenous health, mental health, women’s health and health promotion [34]. Additionally, the Department of Education, with a contribution from the Commonwealth Department of Health, funds allied health therapy services to six state schools in Cape York under the Be well Learn well program. For the Indigenous consumer, this variety of public and non-government providers, funding arrangements and regulatory mechanisms can result in ineffective, non-existent or confusing referral pathways, lower screening rates and limited access [35,36]. There are primary schools in each Cape York community, but there is limited access to secondary education programs.

Palm Island, located 70 kilometres north-east of Townsville, was established in 1918 as a result of colonial social policies which led to the forcible removal and relocation of Indigenous people from 42 other traditional language group areas across Queensland [37]. The estimated resident population is now 2634 [1]. Like Cape York, the population of Palm Island is young and there is a high burden of disease [38]. Queensland Health provides an integrated healthcare service, and a new primary care centre is being constructed. There are two primary schools, and one state secondary school.

### Sampling of schools

The Education Queensland Transition Support Service supports students from Cape York and Palm Island who wish to gain and take up placements and complete their secondary education at boarding schools. Destination boarding schools are based in urban and rural areas of Queensland, with those in southern Queensland being more than 2,000 kilometres from students’ home communities. Boarding schools include Independent Catholic or other Christian schools, state schools, and residential facilities operated under the auspices of sporting organisations. Each school facilitates healthcare to students, with models varying from on-site services to referral out to local primary healthcare or wellbeing services. Boarding schools were included in this study if they met the criteria of having at least 10 Transition Support Service-supported students and falling within one of their three geographical regions (far northern, northern, southern) across Queensland (n = 17 schools).

### Methods

**Figure 2:**
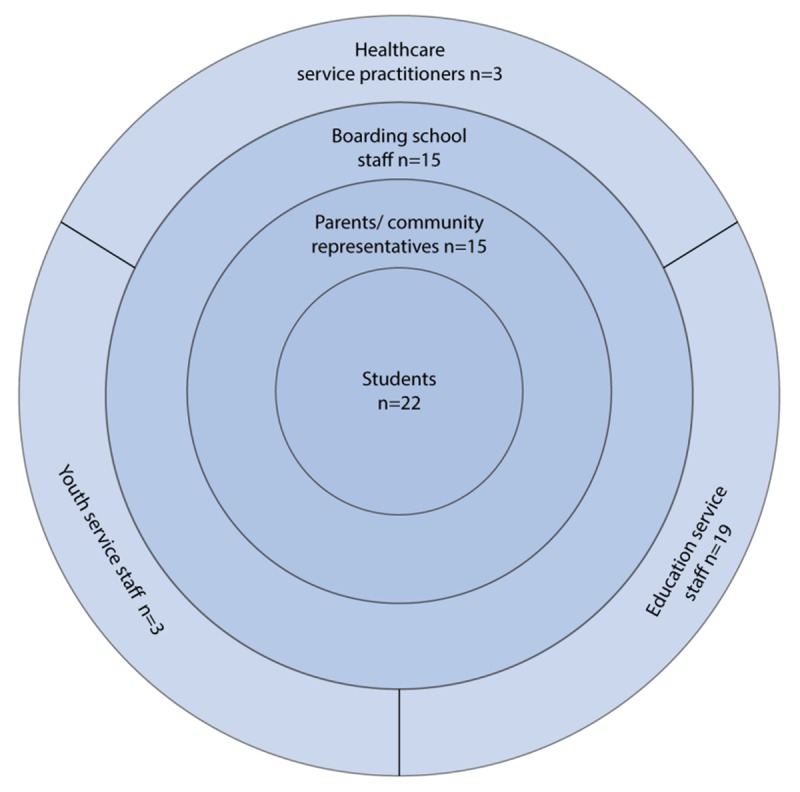
Participants at Schools and Communities Conference.

Consistent with the ecological definition of resilience, we sought feedback on the student survey results from students as well as parents/community members, school staff and healthcare and education service staff. The survey results were presented, with feedback invited, at a two-day Schools and Communities conference held in Cairns in August 2018 (see Figure 2). Students and school staff came from 13 boarding schools across Queensland. Parents and community representatives came from 7 Cape York and Palm Island communities. Healthcare staff were from Apunipima Cape York Health Service; education service staff from the Queensland Department of Education, Catholic Education and Boarding Australia. The survey findings were presented as part of a panel session presented by co-author (JM), with participants asked to give feedback on the findings through two workshops (accessing health services and managing stress).

The accessing health services workshop was facilitated by Indigenous public health practitioner and co-author (MW) and framed to answer the question: How do we ensure continuity of care for students when accessing health care services in community and at school? The managing stress workshop was co-facilitated by an Indigenous public health practitioner and an Indigenous youth worker (DJ and RW) and framed around the three questions: 1) What are key stressors students experience at school? 2) What do students need to move through stressors? 3) Who and what is needed to help students move through these stressors? (Student/Parents/Boarding School/Service Providers). Students and family members were encouraged to contribute to the discussions. Workshop sessions were recorded, transcribed and coded using NVIVO qualitative software. Themes related to each of the research hypotheses were identified using qualitative thematic analysis methods (Braun and Clarke, 2006).

School principals were approached by Transition Support Services and/or Central Queensland University researchers, and all consented to participation of their school in the study. Participating schools signed a Memorandum of Understanding to clarify the expectations and partnership. Informed consent was given in two stages: initial consent was provided by each participant’s parent or guardian, then students also provided informed consent before they began the survey. Participating students named their school and date of birth (to allow re-identification and response to those with high levels of distress), but responses were otherwise de-identified.

The survey was administered by the same three researchers (TB, KR, AB) across all sites during the school term, between January and May, 2018. To accommodate differences in literacy levels and English as a second language needs, students were given the option of having the survey questions read, and if necessary, explained to them by a researcher. The researchers ensured that consistent explanation and language was used across all sites. Students completed the survey using individual devices (iPads) to access an online data collection platform, Qualtrics. Students were provided with snacks but not incentivised through any other means.

An amended Transition Support Service Student Survey (T4S) instrument [24] was utilised. Amendments included use of the Kessler 6 instead of the Kessler 5 and a reduced number of questions. Only measures relevant to the present analysis are outlined below.

Demographics: Participants’ age, gender, and home community were included.

General Health: Self-reported health was assessed using a standard 5 point Likert scale ranging from Excellent to Poor. Self-rated health has been found to be valid in studies of Indigenous adults, although this is attenuated when English is not the primary language [39]. Neither physical health/ill-health nor health risks were measured since they were beyond the scope of the larger resilience study.

Psychological Distress: Psychological distress was measured using the Kessler 6 scale [40]. Psychological distress measures anxiety and depressive symptoms experienced over the previous 4 weeks. This includes feeling nervous, without hope, restless or jumpy, that everything is an effort, and not being able to be cheered up [4]. The Kessler scale has been used in several Indigenous wellbeing surveys [2,41,42,43]. In the current study, the Cronbach alpha coefficient was .84. Scores were categorised as consistent with guidelines for probability of serious psychological distress if above 19 using standard Australian scoring [44].

Healthcare Service Use: The healthcare service use section of the survey was derived from an adapted Pathways to Youth Resilience Measure [45], a composite of internationally validated scales and sub-scales measuring supports available to students and patterns of service use. Adaptations entailed changes to wording to reflect Australian terminology and reducing the number of questions asked. Service use quantity was measured using an abridged version of the Pathways to Youth Resilience Measure (PYRM) that was read through with the young person. The measure was adapted from the Youth Services Survey and has been designed for use with youth as young as nine years old and the reliability of the original scale was 0.75 for youth. However, the abridged version has not been validated internationally or with remote dwelling Indigenous children [46]. Primary school students were asked to respond yes or no to the statement:

“In the last year, I have been to a doctor or clinic in my community”; and if yes, “In the last year, about how many times have you been to a doctor or clinic in your community? (1–2 times, 4–5 times, or 10 times or more).”

Secondary school students were asked the same questions regarding healthcare services both within their home community and at boarding school. Secondary students were also asked whether they had attended other healthcare services for mental health issues, alcohol or drug use, violence or any other wellbeing issues.

Healthcare Service Satisfaction: The healthcare service satisfaction section of the survey was also derived from the adapted Pathways to Youth Resilience Measure [45]. All students were asked their level of satisfaction with the most recent visit to a healthcare service in their home community and while at boarding school.

With a separate informed consent process, conference participants at a Schools and Communities Conference were asked to give feedback on the survey findings through two workshops (accessing healthcare services and managing stress).

### Analysis

All quantitative analyses were conducted in SPSS version 24 (IBM, SPSS Inc). The Kessler 6 scale was checked for internal consistency, and initial descriptive statistics were calculated. Chi square tests for independence were run to examine differences based on either gender or age where appropriate. Qualitative data from the Conference workshops were audiotaped, transcribed verbatim and coded in NVIVO 10. A thematic analysis was conducted of the text data [47].

## Results

Our study engaged a purposive sample of 188 Indigenous secondary boarding school students supported by Transition Support Service, and 32 Indigenous primary school students from two remote communities. A further 16 students who were eligible did not complete the survey (7 chose not to complete the survey, 2 were absent from school due to illness, 3 were absent due to sorry business (bereavement), and 4 were away with sporting commitments). Non-participants were of varied age and gender and there was no pattern to non-completion. Participant characteristics are summarised below at Table 1.

**Table 1:** Participant Age and Gender Breakdown (count).

	Male	Female	Total

**Primary School**			

10 years	2	10	12
11 years	10	8	18
12 years	1	1	2
**Total**	**13**	**19**	**32**
**Secondary School**			

12 years	4	21	25
13 years	16	34	50
14 years	16	13	29
**12–14 years Total**	**36**	**68**	**104**
15 years	12	16	28
16 years	15	18	33
17 years and above	14	9	23
**15 years and above Total**	**41**	**43**	**84**
**Total**	**77**	**111**	**188**

### Self-assessed health status

Two thirds (65.6%) of the 32 primary school students rated their health as excellent or very good and a third (34.4%) as either good or okay (Table 4). No primary age students rated their health as being poor. The small numbers of primary school students make it inappropriate to make claims about statistical significance or generalisations.

Most of the 188 secondary students rated their health as being excellent or very good (72.8%), about a quarter (26.6%) as either good or okay, and very few (0.5%) as poor (Table 2). Self-rated health was relatively consistent across age groups, but female students tended to rate their health better than male students. Having self-rated excellent or very good health was not related to attendance at a healthcare service, either within the community or at boarding school (χ^2^ (1, n = 188) = 1.13, p = .28, phi = –.077 and χ^2^ (1, n = 188) = .221, p = .64, phi = –.039).

**Table 2:** % Distribution of self-rated health by age group and gender (count).

	Excellent	Very Good	Good	Okay	Poor

**Primary School (n = 32)**					

Male	38.5 (5)	30.8 (4)	15.4 (2)	15.4 (2)	0 (0)
Female	36.8 (7)	26.3 (5)	21.1 (4)	15.8 (3)	0 (0)
**Total**	**37.5 (12)**	**28.1 (9)**	**18.8 (6)**	**15.6 (5)**	**0 (0)**
**Secondary School (n = 188)**					

12–14 years					
Male	25.0 (9)	41.7 (15)	19.4 (7)	11.1 (4)	2.8 (1)
Female	45.6 (31)	32.4 (22)	14.7 (10)	7.4 (5)	0 (0)
**Total**	**38.5 (40)**	**35.6 (37)**	**16.3 (17)**	**8.7 (9)**	**1.0 (1)**
15 years and above					
Male	34.1 (14)	34.1 (14)	26.8 (11)	4.9 (2)	0 (0)
Female	37.2 (16)	3.7.2 (16)	23.3 (10)	2.3 (1)	0 (0)
**Total**	**35.7 (30)**	**35.7 (30)**	**25.0 (21**	**3.6 (3)**	**0 (0)**
**Total**	**37.2 (70)**	**35.6 (67)**	**20.2 (38)**	**6.4 (12)**	**0.5 (1)**

#### “When behaviours are normalised, there’s no acknowledgement”

The finding that most students perceived that they had excellent or very good health was considered by Schools and Communities Conference participants to potentially be a result both of protective factors such as large networks of pro-social relationships and a generally optimistic perception of their own health and wellbeing, but also a normalisation of long-term health conditions. An Indigenous education coordinator commented:

“You can’t expect a child to say ‘oh there’s something wrong with me. I should go find [a doctor] …’. When the behaviours are normalised, then – there’s no acknowledgement.”

Participants at the Conference considered that it was unclear whether students’ perceptions of their own health status included both physical and mental health. One (graduated) Indigenous student spoke of her personal health issue:

“I suffered like mental health issues like you know, anxiety, depression … So yeah it was hard being away from your own town and community.”

#### Psychological distress

Only 6.3% of primary school students had high or very high scores for psychological distress over the previous 4 weeks. However, this represented only 2 of the 31 students, one of each gender (Table 3).

**Table 3:** % Risk for psychological distress by age group and gender (count).

	Male	Female	Total

**Primary School (n = 32)**			

**Total**	**7.7 (1)**	**5.3 (1)**	**6.3 (2)**
**Secondary School (n =188)**			

12 years	25.0 (1)	23.8 (5)	24.0 (6)
13 years	12.5 (2)	41.2 (14)	32.0 (16)
14 years	12.5 (2)	30.8 (4)	20.7 (6)
**12–14 years Total**	**13.9 (5)**	**33.8 (23)**	**26.9 (28)**
15 years	25.0 (3)	12.5 (2)	17.9 (5)
16 years	33.3 (5)	33.3 (6)	33.3 (11)
17 years and above	21.4 (3)	44.4 (4)	30.4 (7)
**15 years and above Total**	**26.8 (11)**	**27.9 (12)**	**27.4 (23)**
**Secondary School Total**	**20.8 (16)**	**31.5 (35)**	**27.1 (51)**

For the secondary cohort, the rate of risk of psychological distress was more than four times higher, with just over one quarter of secondary students (27.1%) at high risk for psychological distress. Gender differences were apparent in secondary school students (males 20.8% and females 31.5%), but a chi-square test for independence (with Yates’ Continuity Correction) indicated this was not a significant association: χ^2^ (1, n = 188) = 2.43, p = .14, phi = –.12. Whilst there was some variation between individual ages, a collapsed grouping (due to small cell numbers) of younger (12–14 years) and older (15–18 years) students found these differences were also not significant: χ^2^ (5, n = 188) = 3.32, p = .65, phi = –.13. There was no significant association between high psychological distress and perceptions of poor health; nor between psychological distress and reported attendance at a service for mental health issues, alcohol, drugs, violence or other wellbeing issues in the last 12 months: χ^2^ (1, n = 188) = .01.28, p = .26, phi = –0.1.

The finding of high levels of psychological distress, particularly in the boarding school environment, raised concerns among Conference participants of the potential that schools might fail to identify need and know when to refer appropriately in response. One Indigenous boarding school coordinator, for example, said:

“All of us have the same issues around how do you identify students that really need help, because often they just don’t talk much… they’ll go quiet or tear up a little bit you know, but not really want to go there. …There’s something going on, you know, and it might be big and it might be small, and you just have no idea.”

A holistic perspective was critically important to understanding students’ health issues. An Indigenous healthcare practitioner commented:

“It’s not about what’s happening in front of you, it’s what happened during their childhood and growing up that’s really important.”

A graduate student noted the value of having a close relationship with a school staff member. He said:

“there was a particular staff in the school …I really look up to as a mother. She really cared about– someone I trusted, someone who I went to for advice, someone who cared for me and really gave me that love and care while I was away from home. I really thank her for that.”

### Fragmentation of healthcare services through home community and/or school-provided or referred healthcare services

Three quarters (75%) of primary school students visited a doctor in their home community in the last 12 months, with a higher proportion of males than females attending. About one third of the primary students who used healthcare services did so more than 10 times. A full breakdown of attendance at community healthcare services is shown at Table 4.

**Table 4:** % Use of healthcare service by primary students by gender (count).

	Male	Female	Total

Did not attend	15.4 (2)	31.6 (6)	25.0 (8)
1 or 2 visits	15.4 (2)	21.1 (4)	18.8 (6)
3–5 visits	53.8 (7)	15.8 (3)	31.3 (10)
6–10 visits	0 (0)	0 (0)	0 (0)
10 or more visits	15.4 (2)	31.6 (6)	25.0 (8)
**Total**	**13**	**19**	**32**

For the secondary school cohort, 80% visited a doctor either at home or at school in the last 12 months. Overall rates were relatively consistent across age groups and genders. A chi-square test for independence (with Yates’ Continuity Correction) indicated no significant association between gender and having seen a doctor in the last 12 months: χ^2^ (1, n = 188) = .001, p = .98, phi = –.02. Because of small cell numbers in individual ages, a collapsed grouping of younger (12–14 years) and older (15–18 years) students showed no actual difference in the proportion of younger and older students that visited a doctor in the last twelve months (79.8% for each group).

Two thirds (68.6%) of secondary boarding school students visited a doctor through a home community and a similar proportion (67.8%) visited a doctor through a school-related healthcare service during the past 12 months (Table 5). Over the previous year, 12.2% of secondary students also reported attending a service for mental health issues, alcohol, drugs, violence or other wellbeing issues.

**Table 5:** % Use of healthcare service by secondary students by age group and gender (count).

	Males			Females			Total		

	≤14 years	≥15 years	Total	≤14 years	≥15 years	Total	≤14 years	≥15 years	Total

Attended any health service	83.3 (30)	78.0 (32)	80.5 (62)	77.9 (53)	81.4 (35)	79.3 (88)	79.8 (83)	79.8 (67)	79.8 (150)
Attended community health service									
Did not attend	25.0 (9)	39.0 (16)	32.5 (25)	27.9 (19)	34.9 (15)	30.6 (34)	26.9 (28)	36.9 (31)	31.4 (59)
1 or 2 visits	44.4 (16)	34.1 (14)	39.0 (30)	38.2 (26)	46.5 (20)	41.4 (46)	40.4 (42)	40.5 (34)	40.4 (76)
3 to 5 visits	19.4 (7)	17.1 (7)	18.2 (14)	19.1 (13)	9.3 (4)	15.3 (17)	19.2 (20)	13.1 (11)	16.5 (31)
6 to 10 visits	0 (0)	2.4 (1)	1.3 (1)	5.9 (4)	4.7(2)	5.4 (6)	3.8 (4)	3.6 (3)	3.7 (7)
10 or more visits	11.1 (4)	7.3 (3)	9.1 (7)	8.8 (6)	4.7 (2)	7.2 (8)	9.6 (10)	6.0 (5)	8.0 (15)
Attended school health service									
Did not attend	25.0 (9)	31.7 (13)	33.3 (22)	14.7 (10)	32.6 (14)	31.2 (24)	18.3 (19)	32.1 (27)	32.2 (46)
1 or 2 visits	52.8 (19)	51.2 (21)	51.9 (40)	42.6 (29)	30.2 (16)	40.5 (45)	47.1 (49)	44.0 (37)	45.2 (85)
3 to 5 visits	16.7 (6)	7.3 (3)	11.7 (9)	33.8 (23)	20.8 (11)	30.6 (34)	26.9 (28)	16.7 (14)	22.9 (43)
6 to 10 visits	0 (0)	0 (0)	0 (0)	0 (0)	0 (0)	0 (0)	0 (0)	0 (0)	0 (0)
10 or more visits	5.6 (2)	9.8 (4)	7.8 (6)	8.8 (6)	3.8 (2)	7.2 (8)	7.7 (8)	7.1 (6)	7.4 (14)

#### Over or under servicing?

Despite the recommendation of the National Guide [8] for annual screening of all Indigenous adolescents, an Indigenous healthcare practitioner at the Schools and Communities Conference considered that the use by 75% primary and 81% secondary school students’ overall as:

“probably about the appropriate kind of level of access … This time of life is the healthiest time for Aboriginal and Torres Strait Islander people in their lives… so everyone should be healthy….”

Furthermore, a non-Indigenous education support service provider iterated that even if the level of healthcare use was inadequate, this was not the responsibility of students themselves, but of the adults who care for them. He said:

“Very few kids will access a service of their own volition… In the school system, they are referred, and a small number of kids have got the self-efficacy to refer themselves … We as adults can’t just say the kids aren’t accessing the service…we’re not doing the referrals.”

Participants at the conference were concerned that there were gaps in access to healthcare services through schools that affected use. One Indigenous education support service provider commented:

“services are just not meeting the demands that we have in our respective school communities. And we know that the wellbeing of students is pivotal to their academic success.”

A non-Indigenous boarding school teacher concurred:

“We’ve got a hundred and thirty kids in boarding – all Indigenous. And we’re just scratching the surface with the services that we are able to offer them, and I think we do lose students because we just don’t meet their needs in this area.”

Another Indigenous boarding school staff member commented on the difficulty of accessing mental healthcare and wellbeing services for students when needed. He said:

“Trying to find and access mental health [services] in (name of town where school is located) is very hard. Especially if you’re Indigenous.”

A non-Indigenous primary school teacher from a remote community also considered that the level of healthcare use by students with complex healthcare needs was likely to be insufficient. She reflected:

“we often find that kids present with quite a variety of challenging needs and then they don’t often fit the referral mould for those services….”

The transition of remote-dwelling students to boarding makes it particularly challenging to track the provision of healthcare services. An Indigenous healthcare practitioner noted:

“When our children from Cape York come down to schools … at the moment I’d have to say it’s pretty ad hoc … not particularly systematic how we take care of children ….”

A non-Indigenous primary school teacher observed:

“If we’re building them up and having them work with speech therapists or psychologists or with Child Youth Mental Health through this significant period of the transition and change, how do we continue that at our destination schools? What services are linked in and how do we support and work with boarding schools to make sure that information is shared both ways? We often find that kids are coming back and we don’t know any of the supports that have been put in place at a boarding level and how we can continue that support.”

The Indigenous healthcare practitioner then questioned:

“Are we actually supporting the right students or are [there] students … that have real needs and not actually getting the right services… Sort of over-servicing in some cases and under-servicing in others?”

Barriers to school-based healthcare service provision and linkages included the availability of culturally appropriate services. In seeking support for some mental health challenges experienced while she attended boarding school, one Indigenous graduate commented that it had been challenging to attend a service in which she perceived the practitioner lacked awareness or understanding of Indigenous issues or the holistic Indigenous view of wellbeing. Another barrier for school staff lay in their confusion about which of the government-provided, community-controlled or non-government healthcare services to refer a student to. One Indigenous education coordinator said:

“it’s very hard to know which service to access …So that’s very complex over on Palm. It does add to confusion with us when we’re trying to work out what’s the best way forward for a student and their family.”

An Indigenous boarding school coordinator commented that in the absence of strong relationships with families, obtaining parental permission for healthcare management was difficult:

“So you have to go with your contacts through the school or the Council and say, ‘…look, we need such and such. Give us this’ – and then send off the fax and then unfortunately by the time that it’s happened, maybe they’ve missed one appointment and we’ve had to make it again and then it just rolls on.”

### Healthcare service satisfaction

Primary school students were all either very satisfied or satisfied with their most recent visit to a doctor in their home community healthcare service in the last 12 months, with male students scoring very satisfied more often than females (Table 6). Secondary students also reported high levels of satisfaction. Rates of satisfaction with services were fairly consistent between genders. A higher proportion of younger than older students was very satisfied. There was no relationship between self-rated health and satisfaction with a healthcare service either in community or boarding school (χ^2^ (2, n = 188) = 1.43, p = .49, phi = –.105; χ^2^ (2, n = 188) = 3.59, p = .16, phi = –.189).

**Table 6:** % Rates of satisfaction with healthcare service by age group and gender (count).

	Male	Female	Total

**Community Health Service**			

**Primary School (n = 32)**			

Very satisfied	81.8 (9)	53.8 (7)	66.7 (16)
Satisfied	18.2 (2)	46.2 (6)	33.3 (8)
Not satisfied	0 (0)	0 (0)	(0)
**Secondary School (n = 188)**			

12–14 years			
Very satisfied	51.9 (14)	38.8 (19)	43.4 (33)
Satisfied	48.1 (13)	51.0 (25)	50.0 (38)
Not satisfied	0 (0)	10.2 (5)	6.6 (5)
15 years and above			
Very satisfied	36.0 (9)	35.7 (10)	35.8 (19)
Satisfied	56.0 (14)	60.7 (17)	58.5 (31)
Not satisfied	8.0 (2)	3.6 (1)	5.7 (3)
**School Health Service**			

**Secondary School (n = 188)**			

12–14 years			
Very satisfied	51.9 (14)	38.8 (19)	43.4 (33)
Satisfied	48.1 (13)	51.0 (25)	50.0 (38)
Not satisfied	0 (0)	10.2 (5)	6.6 (5)
15 years and above			
Very satisfied	35.7 (10)	24.1 (7)	29.8 (17)
Satisfied	50.0 (14)	65.5 (19)	57.9 (33)
Not satisfied	14.3 (4)	10.3 (3)	12.3 (7)

#### Not understanding the students’ viewpoint

Conference participants considered that school staff often did not know the reasons for students’ satisfaction or dissatisfaction with healthcare services. One Indigenous education coordinator said:

“how do we understand … the student’s point of view? As an adult, I think we tend to just go in and work from an adult point of view, but from the student’s point of view it’s very different.”

#### Identifying effective healthcare integration models

The processes required by both schools and healthcare services for integrating health care to meet the needs of remote-dwelling Indigenous students is complex. Participants at the Conference considered communication and feedback linkages between students, parents, healthcare services and schools to be the key enabler to integrated healthcare service provision for students. An Indigenous healthcare practitioner commented:

“You know if you’ve got a good relationship, you’ll know … who they [are] – where they should be… But that takes time and relationship building and long term teachers, long term staff … which we don’t necessarily always have.”

He acknowledged, however, that

“It can’t just be left to individuals to know everybody. It’s gotta be in the system and we’ve got to work out ways to make sure that none of our students drop through holes.”

The value of interagency groups in remote communities was suggested by a non-Indigenous education policy advisor who said:

“From my experience on [remote Indigenous community name], behaviour becomes normalised. And we actually had to implement we call it a think-tank where every three weeks we actually sat down and looked at kids and tried to jettison for those things that were contextualised around us, to make sure that we hadn’t raised the bar of tolerance.… that was an inter-agency group.”

But even in the geographically distant context of boarding schools, a non-Indigenous primary school teacher commented on the value of the effort made to develop and maintain personal relationships between community members, boarding school education staff, and other sectors. She commented:

“It was actually refreshing to have [name of Indigenous boarding school coordinator] at [name of remote Indigenous community] on Tuesday… from five years ago, to where we are now, we can see that positive impact and that change… it comes back to that more wrap-around support to chase up those different avenues …by taking these small steps, we’re beginning to work on that support.”

Workshop participants also suggested the value of enhancing school and school staff capacity to provide integrated care for students. An Indigenous education coordinator commented:

“I suppose it’s also that building the capacity of the schools and the staff within the schools to be able to work with the health services, but [also] with the students … we find that’s probably where we need the most help is that building capacity… but you can’t keep on sending staff off to [professional development] PD’s all the time. The schools don’t like it. So yeah so just I suppose trying to work out what’s a good way to go.”

The Indigenous health practitioner, however, drew attention to the simple communication that all education and health practitioners could implement. He concluded:

“…you know, some days you just need a hug. Not tablets and everything else – the therapeutic stuff. And I think those are the kind of things that we need to do as humans a lot better, with our students. So not needing to know all of the psycho babble and all that kind of stuff; being nice to people, being respectful of people, supporting people, lifting people up … will go a long way. … really getting feedback from students, parents and schools around how we could do the linkages better. So when people … have needs, that we target that, prioritise that and make sure that the health system and the wellbeing system, mental health system, picks all this stuff up and a continuum of care no matter where our students are.”

Adult participants provided exemplars of models of healthcare provision that they perceived to be effective. An Indigenous boarding school coordinator offered:

“We’re fortunate, we’ve got our doctors on site with our nurses, so we’ve got our own Health Clinic. We also use people from Deadly Choices and they come in and do some wellbeing there.”

A non-Indigenous education policy officer described his experience of the efforts of a home-community based healthcare provider to link closely with a proximal local state school:

“the model there was like two funnels reversed. The narrow ends pointing together so all of the issues around expediency of information back to teachers…. They brought in all your specialists across a range of different areas. They didn’t just meet kids in school. They’d go and do home visits as well. They’d come back to us and if they couldn’t deliver a service of support that that child needed, they’d outsource it and let us know … and then provide us with two to four key strategies that I could use in my classroom.”

Clearly such linkages become more complex when the linkages need to occur across geographically distant boarding schools and home community services. An Indigenous healthcare practitioner also advised that family-centred and trauma-informed approaches were important.

## Limitations

This study has several limitations. First, the questions in the student survey related to all relevant healthcare services used by students in the last 12 months (not necessarily to experiences with a single service provider or organisation) and did not identify the specific healthcare service used (and so information cannot be fed back to them). Furthermore, the questions asked about experiences up to 12 months after the service was provided, which may have affected the accuracy of the student’s recall. Second, in relation to students’ perceptions of their own health status, it was unclear whether they considered health as encompassing both physical and mental health. We did not measure or have access to data on physical health, so were unable to compare perceptions of health with actual physical health conditions. Third, healthcare service use and satisfaction were used as proxy measures of healthcare quality, but it is difficult to identify whether these are the most appropriate measures. Social desirability response bias may also lead students to report greater satisfaction than they actually feel because they believe positive comments are more acceptable to survey administrators, or because they perceive that complaints may lead to unfavourable treatment in the future [16]. Recent mainstream studies [e.g. 48] have found a tenuous link between patient satisfaction and health care quality and outcomes. Finally, the specific context of Cape York and Palm Island students, and Queensland boarding schools and healthcare services means that the results of this study may not be generalisable to other populations of remote-dwelling Indigenous students at boarding schools.

## Discussion

This study reports the perceptions of Indigenous primary and secondary boarding school students from remote communities of their health status, their utilisation of healthcare services in home communities and when at school, and their levels of satisfaction with healthcare services. Our first hypothesis, that most Indigenous students will assess their health as either excellent or very good, was confirmed. We found that students’ self-rated health status was very high, with 65.6% of primary school students and 72.8% of secondary school students rating their health as excellent or very good. This finding is consistent with the national survey finding of slightly higher ratings by remote dwelling young people than the national Aboriginal and Torres Strait Islander health survey of 63% of Indigenous youth (aged 10–24) rating their health as excellent or very good [4]. In contrast to the national survey findings of a decrease in high ratings with age (79% of those aged 10–14, 60% of those aged 15–19, and only 48% of those aged 20–24), this cohort of students were relatively consistent across age groups in rating their health as excellent or very good (72.05% 10–14 year old and 71.45% of 15–19 year olds) [4]. This higher and consistent rating may be due either to social desirability bias or to protective factors at play in remote areas that mitigate health risk, such as family and/or cultural connections.

For secondary boarding school students, the findings suggested that distress is perceived as normal (27.1% reported high levels of psychological distress) and that students do not perceive distress as reducing their overall health status, nor was it reflected in their use of mental healthcare or wellbeing services. The proportion of students with high levels of psychological distress is consistent with those found in other studies; for example, in urban Inala, 26% of Indigenous adolescents (n = 73) [49], and nationally, 30% of Indigenous young people reported high/very high levels of distress [50]. In the boarding school context, however, it is particularly important that students, boarding school staff and parents are aware of the significance of distress on day to day wellbeing and academic participation, and its potential escalation to more severe illness [51]. The apparent lack of awareness raises concerns about the potential that boarding schools may fail to identify need and to know when to refer students appropriately for clinical and/or psychological support.

Our second hypothesis, that more secondary boarding students will use healthcare services provided through their schools than in their home communities, was not supported. Despite students spending 39/52 weeks of each year at school, and healthcare services being generally more readily available in regional and urban settings, 68.6% of secondary students used healthcare services in their home communities and 67.8% through their school. One-quarter of primary and 19% of secondary school students did not report any visit to a doctor in the previous year, despite the recommendation of the national guide to a preventive health assessment that all Indigenous adolescents be screened annually [8]. These findings suggest an imperative for boarding schools to assess their responsibilities for providing school-based or school-linked healthcare services to augment the primary healthcare services that students receive in their remote home communities, and the critical importance of integration between these two sectors. The medical home model outlined in American studies [15], whereby all students receive health care that is continuous, comprehensive, family centered, culturally sensitive, compassionate, coordinated, and provided by a well-trained health care provider, could be tailored for use with Indigenous Australian students [52].

In particular, the finding that despite 27% of secondary students reporting a high Kessler score, only 12% reported use of a service for mental health, alcohol, drugs or violence raises concerns about whether appropriate youth friendly and after hours services are sufficiently available, and whether students are sufficiently engaged in health-promoting activities such as sports and recreation, arts, music and other after-school activities that can improve social and emotional wellbeing [53]. This finding may be related to evidence of a dearth of pathways developed for, or modified to specifically meet the mental healthcare needs of, Indigenous adolescents [20] and/or poor integration between primary healthcare and mental healthcare services [36].

Given that health and wellbeing are requisite to educational participation and achievement, schools could potentially play a greater role in facilitating access to health and wellbeing services. Integration of care between home community-based and school-based services is important to provide optimal care and prevent over- or under-servicing. However, school staff and healthcare practitioners suggested that barriers to integration of home community and school primary healthcare services resided both in the capacity of school and healthcare service staff, and in the linkages between them. The findings of this study support the recommendation of the National Indigenous Health Equality Council’s roundtable for coordinated action across sectors, including health and education, that pointed to a clear need for improvements in integrated approaches between schools and primary healthcare services [14].

Our third hypothesis, that satisfaction with home community-based healthcare services is likely to be higher than with school-based healthcare services, was not supported. We found a high proportion of students that had high levels of satisfaction with healthcare services overall. There was no statistically significant difference between the 93.8% students who were very satisfied or satisfied with home community services and the 91.1% students who were very satisfied or satisfied with school-based healthcare services. We assume that the mild preference for home community services may be related to the familiarity and cultural competence of community-based services with Indigenous adolescents’ healthcare needs. However, we do not know the expectations of the students, nor why 6.2% of students in this study responded that they were not satisfied or not very satisfied. Ideally, healthcare services for Indigenous adolescents should provide: the appropriate amount of care at the right moment in time, delivered in an adolescent-friendly environment and using a trauma-informed, empowering approach; standardised screening tools; systems to record counselling; and referrals to external services with requisite information, care and support that is tailored for adolescents [20,54]. Responsive health services in both remote home community and school-related sites will need to consider the accessibility and their developmental and cultural appropriateness, including coordination across health services sites and health and education sectors [13,52,53]. In our study, decreasing satisfaction with age may be related to increasing awareness with age of experiences of discrimination/racism in the client encounter and/or embarrassment, and lack of trust in the protection of privacy [55]. There is a need for further research to elucidate Indigenous students’ experiences of specific services, which aspects of healthcare they value, and their suggestions for improvement to healthcare service encounters.

The complexity of healthcare service provision by schools and primary healthcare services in response to the healthcare needs of Indigenous boarding school students suggests the need for an integrated approach. There are potential advantages in providing school-based healthcare services as a way to bring health care closer to adolescents; they can provide improved accessibility, equity and responsiveness to adolescents’ needs [56,57]. Nearly half of the adolescents (47%) responding to a WHO global consultation indicated that they would prefer to obtain healthcare services at school [58]. Such integration requires greater attention to the needs of adolescents in Indigenous health policy; something that has, to date, remained largely at the margins, and exploration of frameworks useful for integrating care [12,59].

## Conclusions

This study explored the perceptions of Indigenous remote community primary and secondary boarding school students of their health status, use of healthcare services and their satisfaction with healthcare services. The level of healthcare service use fell short of the national guide recommendation of 100% annual use, suggesting services may not be meeting the needs of all students. Despite students spending 39/52 weeks of each year at school and healthcare services being generally more readily available in the regional and urban settings of schools, a higher proportion of secondary students used healthcare services in their home communities than through schools. This may reflect family or student preferences but suggests that schools could potentially play a greater role in facilitating access to health and wellbeing services. The lack of significant association between students’ high levels of distress and perceived health status raises concerns about the normalisation of stressors in this population, and the potential that boarding schools may fail to identify need and know when or how to build relationships to enhance psychological support and/or refer appropriately to local health services. Finally, we need to know more about Indigenous students’ expectations of healthcare services and why some students are not satisfied (or not very satisfied) to ensure that all Indigenous students are able to access health services should they require them. This study suggests the need for further exploration of the healthcare models used by boarding schools for Indigenous (and other) students, and how healthcare integration with remote community primary healthcare services could be improved.
